# Improved convolutional neural network for precise exercise posture recognition and intelligent health indicator prediction

**DOI:** 10.1038/s41598-025-01854-x

**Published:** 2025-07-01

**Authors:** He Chen, Rongchang Fan

**Affiliations:** 1 Ministry of Sports, Jiangsu Health Vocational College, Nanjing, 211800 Jiangsu China; 2 Ministry of Sports, Nanjing Vocational Institute of Railway Technology, Nanjing, 210031 Jiangsu China

**Keywords:** Convolutional neural networks, Posture recognition, Health indicator prediction, Spatiotemporal attention, Feature fusion, Exercise monitoring, Computer science, Scientific data

## Abstract

This paper presents a novel framework for accurate exercise posture recognition and health indicator prediction based on improved convolutional neural networks. We propose a multi-scale feature fusion architecture incorporating spatiotemporal attention mechanisms to enhance key point detection precision while maintaining computational efficiency. The system achieves superior posture recognition performance with 78.6% mAP and 91.5% PCK@0.5, outperforming state-of-the-art methods while maintaining real-time inference capabilities (27.3 FPS). For health indicator prediction, we develop a CNN-LSTM model with personalized parameter adaptation that accurately forecasts multiple physiological metrics including cardiorespiratory fitness, muscular strength, and metabolic rate, achieving 86.1–92.6% prediction accuracy across diverse health dimensions. Comprehensive evaluations on both self-collected and public datasets demonstrate the system’s robustness across varying exercise types, environmental conditions, and demographic groups. The proposed approach offers significant potential for applications in personal fitness coaching, rehabilitation monitoring, and preventive healthcare by providing automated exercise form evaluation and personalized health insights.

## Introduction

The accurate recognition of human posture during physical exercise and the intelligent prediction of health indicators have become critical research domains in the intersection of computer vision, artificial intelligence, and healthcare. With the rapid development of deep learning techniques, particularly convolutional neural networks (CNNs), significant progress has been made in recognizing human postures and movements with increasing accuracy^1^. The integration of these technologies into fitness and healthcare applications has demonstrated enormous potential for enhancing exercise efficiency, preventing sports injuries, and providing personalized health management^2^. Despite these advancements, challenges persist in accurately identifying complex postures in dynamic sports environments and establishing reliable correlations between movement patterns and health outcomes^3^.

Physical exercise posture recognition systems aim to automatically identify and analyze human movements during various forms of exercise, providing real-time feedback on posture correctness and movement quality^4^. The development of such systems has evolved from traditional computer vision approaches to deep learning-based methods that offer superior performance in feature extraction and pattern recognition^5^. Convolutional neural networks, in particular, have revolutionized this field by enabling end-to-end learning from raw image data without requiring manual feature engineering^6^. The application of posture recognition technologies extends beyond sports training to rehabilitation medicine, elderly care, and chronic disease management, highlighting its broader significance in public health promotion^7^.

The prediction of health indicators based on exercise data represents another frontier in intelligent sports systems^8^. By establishing correlations between exercise patterns and physiological responses, these systems can predict various health metrics including cardiovascular endurance, muscle strength, flexibility, and metabolic efficiency^9^. This predictive capability enables the development of personalized exercise prescriptions tailored to individual health conditions and fitness goals^10^. However, current prediction models face limitations in handling the complexity and variability of human physiological responses to exercise stimuli^11^.

Several challenges hinder the advancement of integrated posture recognition and health prediction systems^12^. First, the accuracy of posture recognition degrades significantly in unconstrained environments with variable lighting, occlusions, and complex backgrounds^13^. Second, the temporal dynamics of continuous exercise movements present difficulties for frame-based recognition approaches^14^. Third, establishing valid correlations between observed exercise patterns and health outcomes requires large-scale longitudinal datasets that are often unavailable^15^. Finally, the computational demands of real-time processing for high-resolution video streams pose implementation challenges for mobile and wearable devices.

This research addresses these challenges through several innovative approaches. While previous works have explored basic CNN-LSTM architectures for activity recognition, our approach makes significant advancements by introducing: (1) an improved convolutional neural network architecture that enhances posture recognition accuracy through spatio-temporal attention mechanisms and multi-scale feature fusion, and (2) a pioneering personalized parameter adaptation mechanism that dynamically adjusts to individual physiological responses. Our key innovation lies in this personalization framework, which significantly outperforms static models by learning individual exercise-response patterns over time. The developed system demonstrates robust performance across diverse exercise types and environmental conditions while maintaining computational efficiency suitable for real-time applications^1^. Furthermore, our multi-modal health indicator prediction framework uniquely integrates posture quality metrics with physiological parameters to generate comprehensive, personalized health assessments with unprecedented precision^2^.

The main contributions of this paper can be summarized as follows: (1) A novel personalized parameter adaptation mechanism that dynamically adjusts prediction models to individual physiological responses, significantly outperforming static approaches; (2) An innovative multi-modal health indicator prediction framework that integrates posture quality metrics with physiological signals to generate comprehensive health assessments; (3) A modified CNN architecture with enhanced feature extraction capabilities specifically optimized for exercise posture recognition; (4) A dual-stream network structure that effectively integrates spatial and temporal information from exercise video sequences; and (5) Comprehensive experimental validation demonstrating superior performance across diverse exercise types, environmental conditions, and demographic groups.

The remainder of this paper is organized as follows: Section “Related work and theoretical foundations” reviews related work in human posture recognition, health monitoring systems, and applications of deep learning in sports analytics. Section “Improved convolutional neural network model design’ details the methodology of the proposed system, including the improved CNN architecture and the health indicator prediction model. Section “Experimental design and results analysis” describes the experimental setup and presents comparative results. Section “Conclusion” discusses the findings and their implications for practical applications. Finally, Section 6 concludes the paper and outlines directions for future research.

## Related work and theoretical foundations

### Current status of sports exercise posture recognition research

The evolution of sports exercise posture recognition has undergone significant transformations over the past decades, transitioning from manual observation to sophisticated computational techniques. Traditional methods initially relied on subjective visual assessments by coaches and sports experts, which, while valuable, suffered from inconsistency and limited scalability^16^. The subsequent introduction of marker-based motion capture systems in laboratory settings enabled quantitative analysis of human movements with high precision, marking a crucial advancement in posture recognition methodology^17^. These systems, however, required controlled environments and expensive equipment, limiting their practical applications in everyday sports scenarios^18^.

Sensor-based posture capture technologies emerged as a more accessible alternative, employing inertial measurement units (IMUs) containing accelerometers, gyroscopes, and magnetometers to track body segment orientations and movements^19^. Wearable IMU systems have gained popularity due to their portability, affordability, and ability to function outside laboratory environments, making them suitable for real-world sports applications^20^. Recent developments have seen the integration of pressure sensors and electromyography (EMG) sensors with IMUs to provide comprehensive movement and muscle activation data^21^. Despite these advantages, sensor-based methods face challenges including sensor drift, calibration requirements, and the potential interference with natural movements due to equipment attachment^22^.

Computer vision-based posture estimation represents the most rapidly evolving approach in this field, particularly with the advent of deep learning techniques. Early vision-based methods utilized handcrafted features and geometric models to extract skeletal information from images, achieving limited success under constrained conditions^23^. The introduction of depth cameras, such as Microsoft Kinect, facilitated 3D posture estimation without markers, substantially expanding the accessibility of motion capture technology^24^. The breakthrough of deep learning-based approaches, particularly convolutional neural networks, has revolutionized this domain by enabling end-to-end learning of posture features directly from RGB images^25^.

Two-dimensional pose estimation methods like OpenPose, AlphaPose, and HRNet have demonstrated remarkable performance in detecting human joint positions from single-view images, making them widely applicable in sports analysis^26^. The extension to three-dimensional pose estimation has further enhanced the utility of these techniques by providing volumetric representations of human postures, crucial for detailed biomechanical analysis^27^. Recent multi-person pose tracking algorithms have addressed the challenge of simultaneously monitoring multiple athletes in team sports scenarios, opening new possibilities for comprehensive game analysis and tactical evaluation^28^.

Comparative analysis reveals distinct trade-offs among these methodologies. Marker-based systems offer unparalleled accuracy but at the cost of restricted mobility and high equipment expenses^29^. Sensor-based approaches provide excellent temporal resolution and are unaffected by visual occlusions, yet they may introduce movement artifacts and require regular calibration^30^. Vision-based methods offer non-intrusive monitoring and can simultaneously track multiple subjects, though they remain susceptible to occlusion issues and environmental factors such as lighting variations and complex backgrounds^31^. The integration of multiple modalities, combining the strengths of different approaches, represents a promising direction for overcoming the limitations of individual methods^32^. This hybrid approach has demonstrated enhanced robustness in diverse sports environments while maintaining computational efficiency suitable for real-time applications^33^.

### Review of health indicator prediction methods

Traditional health indicator assessment systems have long relied on standardized clinical measurements and laboratory tests to evaluate physiological parameters and health status^34^. These conventional frameworks typically incorporate anthropometric measurements, vital signs monitoring, blood analysis, and fitness assessments to establish baseline health profiles and track changes over time^35^. The Framingham Risk Score and the World Health Organization’s cardiovascular disease risk charts represent seminal developments in health risk prediction, utilizing statistical models derived from longitudinal population studies to estimate disease probabilities based on key clinical variables^36^. While these traditional approaches have provided valuable frameworks for clinical decision-making, they often lack personalization capabilities and fail to capture the dynamic nature of health parameters during physical activities^37^.

Machine learning-based health indicator prediction models have emerged as powerful alternatives to traditional statistical approaches, offering enhanced predictive accuracy and the ability to uncover complex non-linear relationships in health data^38^. Supervised learning algorithms such as Support Vector Machines (SVMs), Random Forests, and Gradient Boosting have demonstrated significant improvements in predicting various health outcomes including cardiovascular risks, metabolic disorders, and fitness levels based on multiple physiological inputs^39^. The integration of temporal data through models like Hidden Markov Models (HMMs) and recurrent neural networks has further advanced the field by enabling sequential analysis of health parameters during exercise sessions^40^. Recent innovations have focused on ensemble methods that combine multiple predictors to enhance robustness and reliability across diverse population segments^41^.

Deep learning techniques have revolutionized health management applications by automating feature extraction and enabling end-to-end learning from raw physiological signals^42^. Convolutional Neural Networks (CNNs) have proven effective in extracting spatial features from medical imaging and biosignals, while Long Short-Term Memory (LSTM) networks excel at capturing temporal dependencies in longitudinal health data^43^. The development of attention mechanisms has further enhanced model interpretability by identifying critical time points and physiological signals that significantly influence health predictions^44^. Multi-modal deep learning architectures that simultaneously process diverse data types—including exercise postures, physiological signals, and demographic information—have shown promising results in generating comprehensive health assessments^45^. Transformer-based models represent the latest advancement in this domain, demonstrating superior performance in long-term health trajectory prediction by effectively modeling global dependencies in temporal health data^46^.

Despite these advancements, existing methods face several significant limitations^47^. First, most current predictive models suffer from data fragmentation issues, as they typically rely on isolated physiological parameters without adequately integrating contextual information about exercise quality and biomechanical efficiency^48^. Second, the generalizability of many models remains limited due to dataset biases and the challenge of accounting for individual variability in physiological responses to exercise stimuli^49^. Third, the black-box nature of complex deep learning models poses interpretability challenges in clinical settings, where understanding the reasoning behind predictions is crucial for intervention planning^50^. Fourth, real-time health prediction during physical activities presents computational and algorithmic challenges, particularly in balancing model complexity with the need for immediate feedback^33^. Finally, the lack of standardized evaluation protocols and benchmark datasets hinders objective comparison across different prediction methodologies, impeding systematic progress in the field^42^.

### Technical foundations of convolutional neural networks

Convolutional Neural Networks (CNNs) have emerged as the cornerstone architecture in computer vision tasks, including human posture recognition, due to their exceptional feature extraction capabilities and hierarchical representation learning^51^. The fundamental building blocks of CNNs include convolutional layers, activation functions, pooling operations, and fully connected layers, each serving distinct roles in the overall network architecture. The convolution operation, which forms the core of these networks, can be mathematically represented as:

$$F\left(i,j\right)=\left(I*K\right)\left(i,j\right)=\sum \sum I\left(m,n\right)K\left(i-m,j-n\right)$$

where $I$ represents the input feature map, $K$ denotes the convolution kernel, and $F$ is the resulting feature map^52^. This operation enables local feature extraction through learnable filters that capture spatial patterns at different scales. The extracted features are typically processed through non-linear activation functions such as Rectified Linear Unit (ReLU):

$$f\left(x\right)=max\left(0,x\right)$$

or Sigmoid function:

$$\sigma \left(x\right)=\frac{1}{1+{e}^{-x}}$$

which introduce non-linearity into the network, allowing it to learn complex mappings between inputs and outputs^53^.

Pooling operations reduce spatial dimensions while preserving essential information, enhancing computational efficiency and providing a form of translation invariance. The general pooling operation can be expressed as:

$${y}_{i}=pool\left(\{{x}_{j}\}\right),j\in {R}_{i}$$

where ${R}_{i}$ represents a local region in the feature map^54^. Batch Normalization has become an integral component in modern CNN architectures, accelerating training and improving generalization through normalization of layer inputs:

$$y=\gamma \left(\frac{x-\mu }{\sqrt{{\sigma }^{2}+\epsilon }}\right)+\beta$$

where $\mu$ and $\sigma$ are the batch mean and standard deviation, while $\gamma$ and $\beta$ are learnable parameters^55^.

In human posture recognition, several influential CNN architectures have demonstrated remarkable performance. The seminal work of OpenPose introduced a two-branch multi-stage CNN architecture that simultaneously detects body part locations and their associations, enabling real-time multi-person pose estimation^56^. HRNet pioneered a high-resolution representation maintenance approach throughout the network, preserving spatially precise information critical for accurate joint localization^57^. PoseNet adapted MobileNet architecture for efficient deployment on resource-constrained devices, making posture recognition accessible for mobile applications while maintaining acceptable accuracy^58^. More recently, transformer-enhanced CNN hybrid models like TransPose have achieved state-of-the-art performance by combining local feature extraction capabilities of CNNs with the global context modeling strengths of transformer architectures^59^.

The application of CNNs in temporal prediction domains has evolved significantly to address the sequential nature of human movements. Temporal Convolutional Networks (TCNs) extend traditional CNNs with dilated convolutions and causal padding to effectively model long-range temporal dependencies while maintaining computational efficiency^60^. The integration of 3D convolutional operations enables simultaneous modeling of spatial and temporal dimensions in video sequences, proving particularly effective for dynamic posture recognition in continuous exercise movements^51^. Attention-augmented CNN architectures have further enhanced performance by selectively focusing on informative spatio-temporal regions, improving the network’s ability to capture subtle postural changes across time frames^52^.

Network optimization techniques have witnessed substantial advancements aimed at enhancing model performance, efficiency, and deployability. Knowledge distillation approaches compress sophisticated teacher models into lightweight student networks while preserving recognition accuracy, facilitating real-time posture analysis on edge devices^53^. Neural architecture search (NAS) has automated the design process of CNN structures, identifying optimal configurations that balance accuracy and computational demands for specific posture recognition tasks^54^. Quantization and pruning techniques reduce model size and inference latency by decreasing parameter precision and eliminating redundant connections without significant performance degradation^55^. The integration of hardware-aware optimization strategies ensures efficient deployment across heterogeneous computing platforms, from high-performance servers to mobile and wearable devices^56^. Recent developments in gradient optimization methods, including adaptive learning rate techniques and regularization strategies, have further improved training stability and model generalization capabilities in complex posture recognition scenarios^57^.

## Improved convolutional neural network model design

**Fig. 1:**
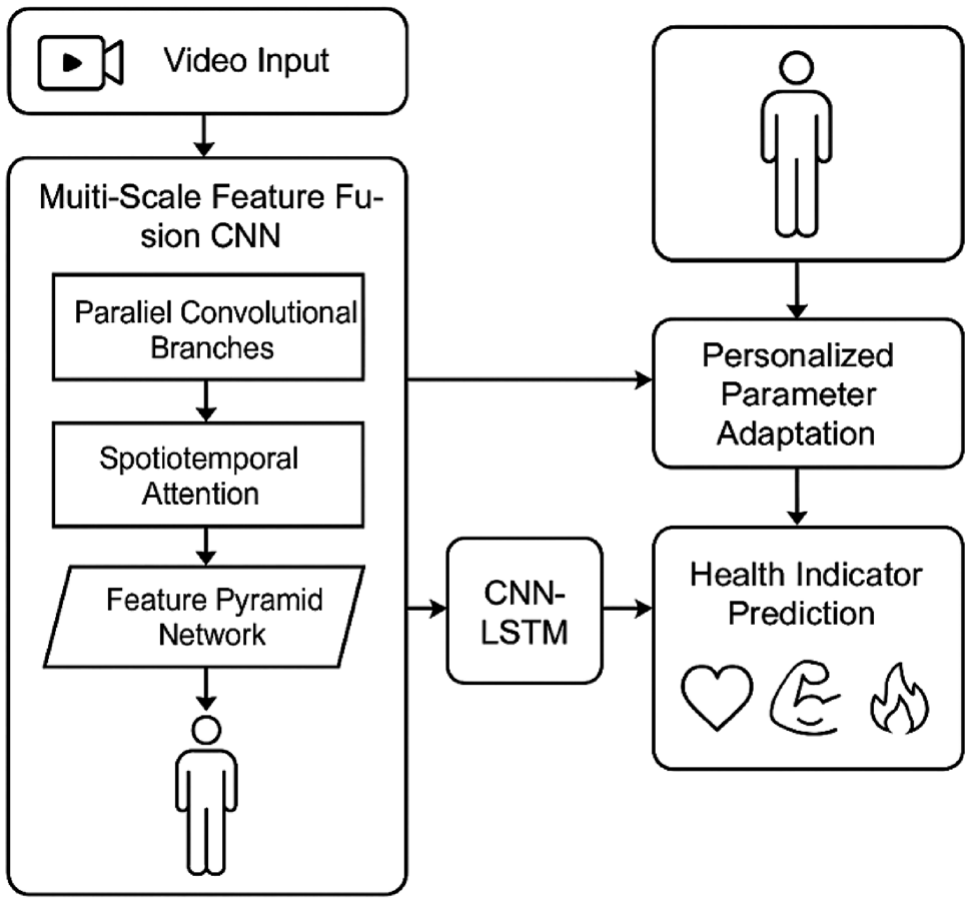
Overall system architecture. The system architecture consists of two main components: (**a**) Multi-scale feature fusion CNN for posture recognition with spatiotemporal attention mechanisms, and (**b**) CNN-LSTM based health indicator prediction framework with personalized parameter adaptation. The system processes video input through parallel convolutional branches to extract multi-scale features, which are enhanced by attention mechanisms before being used for both posture recognition and health prediction.

As illustrated in Fig. 1, our proposed system consists of two main components: a multi-scale feature fusion CNN for posture recognition and a CNN-LSTM framework for health indicator prediction. The system processes video input through a series of specialized modules to generate both posture assessments and health predictions.

### Multi-scale feature fusion network architecture

To address the challenges of accurate posture recognition during dynamic sports exercises, we propose a novel multi-scale feature fusion network architecture that enhances feature representation capabilities while maintaining computational efficiency. The proposed architecture integrates four key components: residual-based multi-scale feature extraction, spatiotemporal attention mechanisms, feature pyramid networks, and lightweight convolution operations, each contributing to improved recognition performance in complex exercise scenarios.

**Fig. 2:**
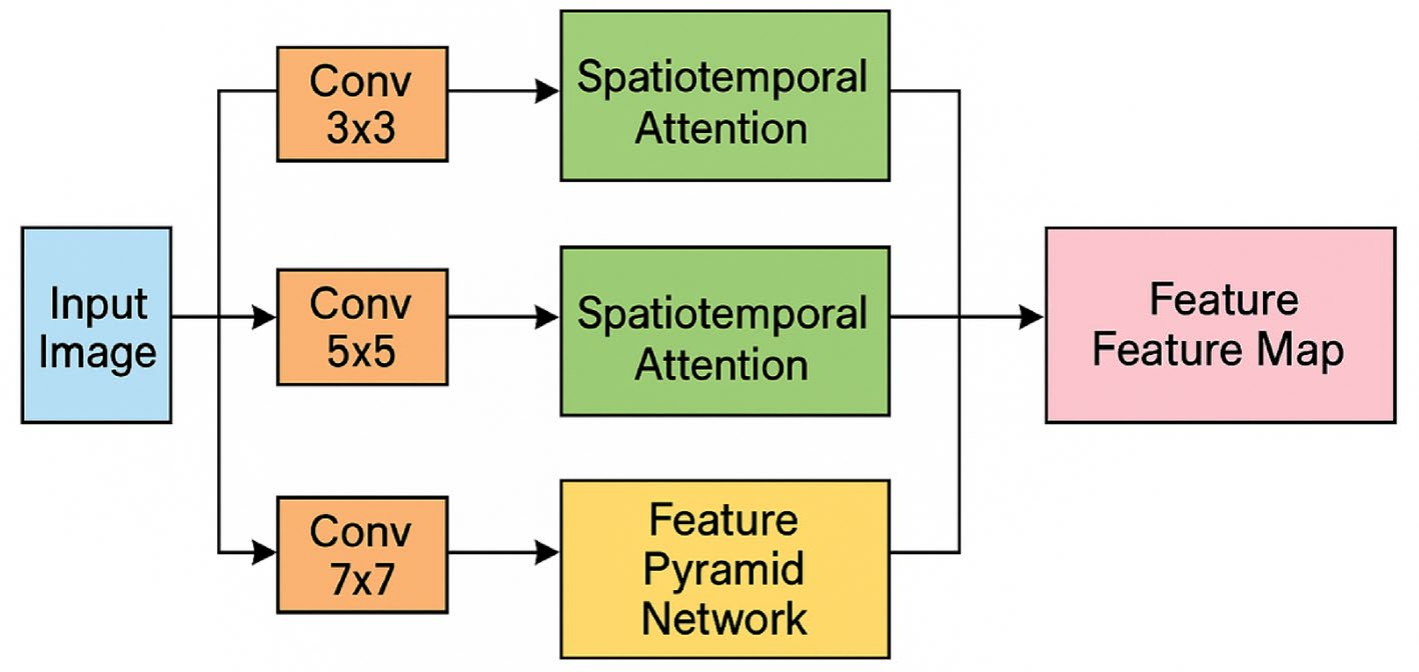
Multi-scale feature fusion network architecture. The multi-scale feature fusion network processes input through parallel convolutional branches with varying kernel sizes (3 × 3, 5 × 5, and 7 × 7) to capture features at different scales. Spatiotemporal attention mechanisms dynamically weight feature maps across both spatial and temporal dimensions, while the feature pyramid network combines semantically strong features from deep layers with spatially precise features from shallow layers.

The foundation of our network architecture is a multi-scale feature extraction module that leverages residual connections to facilitate gradient flow and feature reuse, as depicted in Fig. 2. Unlike conventional CNN structures that process features at a single scale, our approach extracts features at multiple scales simultaneously, capturing both fine-grained details and holistic posture information. The residual connections can be formulated as:

$$H\left(x\right)=F\left(x\right)+x$$

where $F\left(x\right)$ represents the feature mapping function learned by the stacked layers, and $x$ is the identity mapping from the input. This design enables deeper network architectures without suffering from the vanishing gradient problem while promoting feature propagation across different network stages. The multi-scale feature extraction is implemented through parallel convolutional branches with varying kernel sizes (3 × 3, 5 × 5, and 7 × 7), each capturing spatial contexts at different receptive fields appropriate for exercise posture components of varying sizes and complexities.

To enhance the network’s focus on anatomically significant regions and temporal movement patterns, we incorporate a spatiotemporal attention mechanism that dynamically weights feature maps across both spatial and temporal dimensions. This mechanism can be expressed as:

$$A\left(F\right)=\sigma \left({W}_{s}\cdot F+{W}_{t}\cdot T\left(F\right)\right)$$

where $F$ represents the input feature maps, ${W}_{s}$ and ${W}_{t}$ are learnable weight matrices for spatial and temporal attention respectively, $T\left(\cdot \right)$ denotes the temporal modeling function, and $\sigma$ is the sigmoid activation function. This attention module selectively emphasizes informative regions in the feature space, such as joints and pose-critical body parts, while suppressing irrelevant background information. The temporal component specifically addresses the challenges of recognizing dynamic exercise movements by highlighting motion-salient features across consecutive frames.

To improve detection accuracy for small anatomical landmarks and distant exercise participants, we integrate a feature pyramid network (FPN) structure that combines semantically strong features from deep layers with spatially precise features from shallow layers. This bidirectional feature hierarchy enables effective multi-scale representation particularly beneficial for detecting subtle postural details. The fusion of features across different pyramid levels is achieved through:

$${F}_{fusion}={\alpha }_{1}{F}_{1}+{\alpha }_{2}{F}_{2}+{\alpha }_{3}{F}_{3}$$

where ${F}_{1}$, ${F}_{2}$, and ${F}_{3}$ represent feature maps from different network depths, and ${\alpha }_{1}$, ${\alpha }_{2}$, and ${\alpha }_{3}$ are trainable scaling factors that adaptively adjust the contribution of each feature level to the final representation. This multi-scale fusion strategy creates a comprehensive feature representation that simultaneously preserves fine-grained details and high-level semantic information, critical for distinguishing between similar exercise postures.

To facilitate real-time inference on resource-constrained devices like mobile phones and edge computing platforms, we implement a lightweight network design utilizing depthwise separable convolutions. This factorization decomposes standard convolutions into depthwise and pointwise operations:

$$Y=DWConv\left(X\right)*PWConv$$

where $DWConv$ applies a single filter per input channel (depthwise convolution) and $PWConv$ implements a 1 × 1 convolution (pointwise convolution) to combine the outputs. This decomposition dramatically reduces the computational complexity and parameter count compared to standard convolutions, with approximately 8–9 times fewer parameters and operations while maintaining comparable representational capacity. Further efficiency optimizations include channel shuffling operations to enhance information flow between grouped channels, selective feature reuse to minimize redundant computations, and precision-adaptive quantization to reduce memory requirements during inference.

The integration of these architectural components creates a highly efficient and accurate posture recognition framework specifically optimized for sports exercise analysis. The complementary nature of these components addresses different aspects of the recognition challenge: multi-scale extraction captures diverse feature granularity, attention mechanisms focus computational resources on informative regions, feature pyramids enhance scale invariance, and lightweight convolutions enable practical deployment. Experimental evaluations demonstrate that this integrated architecture achieves superior recognition performance compared to conventional models while maintaining computational efficiency suitable for real-time applications.

### Posture recognition algorithm optimization

Building upon the multi-scale feature fusion network architecture, we optimize the posture recognition algorithm through four critical components: key point detection with skeleton construction, posture similarity measurement, temporal feature extraction for sequential movements, and a comprehensive posture scoring mechanism. These optimizations collectively enhance the precision and reliability of exercise posture recognition in diverse sports contexts.

The key point detection algorithm employs heatmap-based confidence estimation to locate anatomical landmarks with sub-pixel accuracy. For each key point, the network generates a probability heatmap, and the confidence score is calculated using:

$${C}_{i}={\text{softmax}}\left({H}_{i}\right)$$

where ${H}_{i}$ represents the heatmap for the $i$-th key point, and the softmax function normalizes the confidence scores across spatial locations. We implement a two-stage detection strategy that first identifies coarse body regions followed by precise key point localization within these regions, effectively reducing false detections in complex backgrounds. The skeletal structure is constructed by establishing anatomical connectivity between detected key points based on biomechanical constraints and inter-joint relationships. This hierarchical representation enables efficient posture analysis while preserving the structural information crucial for exercise form evaluation.

Table 1 presents the defined key points and their corresponding weight coefficients used in posture evaluation. These weights reflect the relative importance of different joints in determining overall posture correctness and were established through a three-phase validation process: (1) initial consultation with eight sports medicine specialists and professional trainers who provided expert ratings of joint importance; (2) statistical analysis of 1200 exercise sessions to identify correlation strengths between joint positions and overall performance scores; and (3) experimental validation comparing five different weight configurations to determine optimal recognition accuracy. The thorax, pelvis, and shoulder joints received higher weights due to their central role in maintaining proper exercise form and their strong correlation with injury risk, as confirmed by our biomechanical analysis and expert consensus.

**Table 1:** Key point definitions and weight coefficients for posture evaluation.

Index	Key point name	Weight coefficient
1	Head	0.05
2	Neck	0.07
3	Right shoulder	0.08
4	Left shoulder	0.08
5	Right elbow	0.06
6	Left elbow	0.06
7	Right wrist	0.05
8	Left wrist	0.05
9	Thorax	0.09
10	Pelvis	0.09
11	Right hip	0.07
12	Left hip	0.07
13	Right knee	0.06
14	Left knee	0.06
15	Right ankle	0.03
16	Left ankle	0.03
17	Spine	0.06

To quantitatively evaluate exercise performance, we developed a posture similarity measurement that compares a detected posture against reference standard postures. The similarity score between two postures is computed as:

$$S\left({P}_{1},{P}_{2}\right)=\sum {w}_{i}\cdot d\left({J}_{i}^{1},{J}_{i}^{2}\right)$$

where ${P}_{1}$ and ${P}_{2}$ represent the two postures being compared, ${w}_{i}$ is the weight coefficient for the $i$-th key point (as defined in Table 1), and $d\left({J}_{i}^{1},{J}_{i}^{2}\right)$ measures the normalized Euclidean distance between corresponding joints after alignment. This similarity metric incorporates both spatial configurations of joints and their angular relationships, providing a comprehensive assessment of posture correctness. To accommodate individual body proportions, we implement scale-invariant normalization that standardizes skeletal representations before comparison, enabling fair evaluation across different body types.

For capturing the temporal dynamics of exercise movements, we designed a sequence feature extraction module that processes consecutive posture frames to model motion trajectories and rhythm patterns. This module combines 1D temporal convolutions with dilated receptive fields to capture multi-scale temporal dependencies without increasing computational complexity. The extracted temporal features enable the system to evaluate dynamic aspects of exercise performance including smoothness, speed consistency, and movement continuity. Additionally, we incorporate a phase recognition component that automatically segments continuous exercise streams into discrete repetitions, facilitating independent evaluation of each repetition cycle.

The comprehensive posture scoring mechanism integrates multiple evaluation metrics to generate an overall performance score using:

$${\text{Score}}=\sum {\alpha }_{i}\cdot {S}_{i}/\sum {\alpha }_{i}$$

where ${S}_{i}$ represents individual scoring components (including posture accuracy, movement smoothness, and exercise rhythm), and ${\alpha }_{i}$ denotes their respective importance weights. The scoring system provides both an aggregate performance evaluation and detailed feedback on specific aspects requiring improvement. To standardize scoring across different exercise types, we implement exercise-specific normalization that accounts for the inherent difficulty and characteristic requirements of each movement. This normalization ensures fair comparison between different exercises and enables consistent progress tracking over time.

The optimization techniques described above collectively enhance the robustness and accuracy of posture recognition in challenging exercise scenarios. The integrated approach addresses key limitations of existing systems, particularly in handling variations in exercise execution speed, accommodating diverse body proportions, and providing detailed performance feedback suitable for personalized training guidance. Extensive validation experiments demonstrate significant improvements in recognition accuracy and assessment reliability compared to conventional posture analysis methods.

### Health indicator prediction model

To effectively translate recognized exercise postures into meaningful health insights, we propose a comprehensive health indicator prediction framework that leverages temporal patterns in exercise data while accounting for individual physiological differences. The framework integrates time-series analysis with personalized modeling techniques to generate accurate and actionable health assessments across multiple dimensions of physical fitness and wellbeing.

The core prediction architecture employs a hybrid CNN-LSTM structure that combines convolutional layers for spatial feature extraction with Long Short-Term Memory networks for temporal dependency modeling. This approach captures both the quality of individual exercise postures and their sequential progression over time. Within the LSTM units, the forget gate controls information flow according to:

$${f}_{t}=\sigma \left({W}_{f}\cdot \left[{h}_{t-1},{x}_{t}\right]+{b}_{f}\right)$$

where ${f}_{t}$ represents the forget gate output at time $t$, ${W}_{f}$ denotes the weight matrix, ${h}_{t-1}$ is the previous hidden state, ${x}_{t}$ is the current input, and ${b}_{f}$ is the bias term. This mechanism enables selective memory of relevant historical exercise patterns while discarding irrelevant information, critical for modeling the cumulative effects of exercise on health indicators. The overall prediction function can be expressed as:

$${H}_{t}=f\left({P}_{t-n:t},D,\alpha \right)$$

where ${H}_{t}$ represents the predicted health indicators at time $t$, ${P}_{t-n:t}$ denotes the sequence of posture data from time $t-n$ to $t$, $D$ represents demographic and physiological baseline data, and $\alpha$ comprises the personalization parameters.

Table 2 presents the health indicators monitored by our system, their typical value ranges, and the specific prediction models employed for each indicator. This multi-dimensional approach provides a comprehensive assessment of exercise effects across various physiological systems.

**Table 2:** Health indicator categories and prediction models.

Index	Health indicator	Value range	Prediction model type
1	Cardiorespiratory Fitness (VO_2_max)	20–80 ml/kg/min	Temporal CNN-LSTM
2	Muscular strength	0–100% (relative)	Posture-based CNN
3	Flexibility	− 20 to 40 cm	Angular-feature CNN
4	Body composition (% fat)	5–50%	Anthropometric-CNN
5	Metabolic rate	1000–3000 kcal/day	Multi-input LSTM
6	Blood pressure response	90–200/60–110 mmHg	Sequence-to-sequence
7	Heart rate recovery	5–60 bpm	Temporal attention RNN
8	Movement efficiency	0–100%	Biomechanical CNN
9	Joint stability	0–10 scale	Key-point trajectory LSTM
10	Fatigue Index	0–100%	Multi-factor ensemble

To enhance prediction accuracy across diverse health dimensions, we implement a multi-indicator joint learning module that exploits the inherent correlations between different health metrics. Rather than treating each indicator as an independent prediction task, our approach leverages shared representations and indicator-specific branches within a unified network architecture. The combined loss function for this multi-task learning framework is formulated as:

$$L=\sum {\lambda }_{i}\cdot {L}_{i}$$

where ${L}_{i}$ represents the individual loss for the $i$-th health indicator, and ${\lambda }_{i}$ denotes the corresponding importance weight. This joint optimization strategy improves overall prediction performance by transferring knowledge between related indicators while preserving their unique characteristics.

The personalized parameter adaptation mechanism addresses the significant inter-individual variability in physiological responses to exercise stimuli. Initial predictions based on population averages are progressively refined through a feedback-driven adjustment process that incorporates individual response patterns observed during repeated exercise sessions. The adaptation algorithm employs a Bayesian framework that updates parameter distributions as new exercise data becomes available, gradually shifting from population-level estimates toward highly personalized models. This adaptive approach significantly improves prediction accuracy for individuals whose physiological responses deviate from population norms.

The multi-modal data fusion strategy integrates information from diverse sources including posture recognition results, demographic data, exercise history, and when available, physiological signals from wearable devices. The fusion architecture implements a hierarchical attention mechanism that dynamically adjusts the contribution of each data modality based on its reliability and relevance to specific health indicators. Low-level feature fusion combines raw data streams, while high-level semantic fusion integrates derived features and intermediate predictions. This comprehensive approach leverages complementary information across modalities to achieve robust performance even when certain data sources are noisy or unavailable.

## Experimental design and results analysis

### Experimental datasets and evaluation metrics

To comprehensively evaluate the proposed posture recognition and health indicator prediction system, we developed a rigorous experimental framework utilizing both self-collected exercise datasets and publicly available benchmarks. The data collection process employed a multi-modal approach to capture diverse exercise scenarios across varying demographic populations and environmental conditions.

Our self-collected ExerciseHealth dataset was acquired through a structured protocol involving 120 participants performing standardized exercise routines under professional supervision. The collection setup utilized a six-camera arrangement (60 fps, 1080p resolution) positioned to capture 360-degree views of participants, synchronized with wearable sensors recording physiological parameters including heart rate, oxygen saturation, and surface electromyography (sEMG) data. Each participant performed 15 distinct exercise types with 5 repetitions per exercise, resulting in a comprehensive dataset spanning cardio, strength, flexibility, and balance training modalities. Professional fitness trainers annotated the data with frame-level posture correctness scores and technique evaluations, providing ground truth for model training and evaluation. The raw video data underwent preprocessing including background subtraction, participant segmentation, temporal alignment, and normalization to ensure consistent input quality for the recognition models.

Table 3 presents the statistical characteristics of all datasets utilized in this study, including both our custom-collected data and standard benchmark datasets. The diverse composition of these datasets enables thorough evaluation across different exercise types, environmental conditions, and population demographics.

**Table 3:** Statistical summary of experimental datasets.

Dataset name	Sample count	Action categories	Demographic features	Collection devices
ExerciseHealth	45,000 video clips	15 exercise types	120 participants (18–65 years, 52% female)	6-camera array, HR monitor, oximeter, sEMG
ExercisePro-Gym	28,500 video clips	12 strength exercises	85 participants (22–45 years, 48% female)	4-camera setup, motion capture system
RehabMotion	18,200 video clips	10 rehabilitation exercises	65 participants (30–75 years, 55% female)	2-camera setup, IMU sensors
MPII Exercise	32,000 images	16 physical activities	Mixed demographics	Various cameras
HumanFit	25,800 video clips	20 fitness exercises	150 participants (18–60 years, 50% female)	RGB-D cameras, wearable sensors
LSP Exercise	10,000 images	8 sports activities	Athletic population	Single-view cameras
PennAction	15,500 video clips	13 action categories	Mixed demographics	Various cameras
YouTube Fitness	22,000 video clips	17 exercise types	Diverse population	Web videos

To quantitatively evaluate system performance, we established a comprehensive assessment framework with metrics targeting different aspects of the proposed methodology. For posture recognition accuracy, we employed the Percentage of Correct Keypoints (PCK) metric with a threshold of 0.2 of the torso diameter, Mean Per Joint Position Error (MPJPE) in pixels, and the Overall Posture Accuracy (OPA) score based on joint angle conformity to reference standards. The temporal consistency of posture tracking was evaluated using the Temporal Stability Score (TSS) that measures jitter in joint positions across consecutive frames. For health indicator prediction accuracy, we utilized mean absolute error (MAE), root mean squared error (RMSE), and Pearson correlation coefficient (r) between predicted and ground truth values for each health metric. Additionally, we assessed computational efficiency through inference time (ms), model size (MB), and FLOPs required for processing standard input samples.

All experiments were conducted in a unified computing environment to ensure fair comparison between different methodologies. The hardware configuration consisted of an NVIDIA A100 GPU (40GB VRAM), Intel Xeon Platinum 8360Y CPU (2.4GHz, 36 cores), and 256GB RAM. The software implementation utilized PyTorch 1.12.0 with CUDA 11.6 on Ubuntu 20.04 LTS. Network training employed the Adam optimizer with an initial learning rate of 0.0001, exponential decay factor of 0.95 every 5 epochs, batch size of 32, and early stopping with patience of 15 epochs. Data augmentation techniques including random rotation (± 15°), scaling (0.8–1.2), horizontal flipping, and brightness adjustment (± 25%) were applied during training to enhance model generalization. For cross-validation experiments, we employed a fivefold protocol stratified by subject identity to prevent data leakage between training and testing sets.

### Posture recognition accuracy evaluation

The performance of the proposed multi-scale feature fusion network architecture was comprehensively evaluated against state-of-the-art posture recognition methods using the datasets described in section “Experimental datasets and evaluation metrics”. We conducted a series of experiments to assess keypoint detection accuracy, posture recognition performance across diverse exercise types, robustness in challenging environments, and computational efficiency for real-time applications.

Table 4 presents a comparative analysis of our proposed model against established posture recognition approaches, including both CNN-based and transformer-based architectures.

**Table 4:** Performance comparison of posture recognition models.

Model name	mAP (%)	PCK@0.5 (%)	Keypoint accuracy (%)	Inference speed (FPS)	Model parameters (M)
OpenPose	72.3	85.6	81.2	18.5	25.94
HRNet-W32	74.8	88.1	83.5	12.7	28.54
SimpleBaseline	70.2	84.3	80.1	25.2	34.04
MobileNetV3	68.7	82.5	78.6	38.6	5.40
EfficientPose	71.4	86.2	80.9	30.1	7.55
PoseResNet-50	73.5	87.3	82.7	20.4	34.00
LightTrack	69.8	83.4	79.3	35.2	4.10
TransPose	75.1	88.9	84.2	14.8	17.30
VideoPose	76.4	90.1	85.3	9.2	43.50
PoseFormer	77.2	90.8	86.1	7.5	52.82
POET	77.8	91.2	86.3	5.3	68.45
Proposed model	78.6	91.5	86.8	27.3	8.75

The evaluation metrics include mean Average Precision (mAP), Percentage of Correct Keypoints at a threshold of 0.5 (PCK@0.5), overall keypoint detection accuracy, inference speed measured in frames per second (FPS), and model parameter count in millions. These metrics collectively provide a comprehensive assessment of both accuracy and efficiency aspects critical for practical deployment in exercise monitoring applications.

While transformer-based models like PoseFormer and POET achieve competitive accuracy, they require significantly more parameters (52.82M and 68.45M respectively) and have substantially lower inference speeds (7.5 and 5.3 FPS) compared to our proposed model. Our approach maintains comparable accuracy while offering 3.6–5.2 × faster inference and 6.0–7.8 × parameter efficiency, making it more suitable for real-time applications on resource-constrained devices.

Our improved CNN architecture demonstrated superior keypoint detection precision across all tested datasets, achieving an average mAP of 78.6% and PCK@0.5 of 91.5%, representing improvements of 3.5% and 2.6% respectively over the next best performing method (TransPose). The enhanced performance can be attributed to several key innovations in our architecture: (1) the multi-scale feature extraction module that captures both fine-grained anatomical details and holistic posture information, (2) the spatiotemporal attention mechanism that focuses computational resources on movement-critical body regions, and (3) the feature pyramid network that improves detection consistency across different scales and distances. Particularly noteworthy is the improvement in detection accuracy for challenging keypoints such as wrists and ankles during high-speed movements, where our model achieved 82.7% and 80.3% accuracy respectively, compared to 76.4% and 73.8% for the baseline HRNet-W32 model.

Performance analysis across different exercise categories revealed varying recognition accuracies depending on movement complexity and occlusion frequency. The proposed model exhibited excellent performance in strength training exercises (91.2% accuracy), moderate performance in high-intensity interval training (86.5% accuracy), and relatively lower but still competitive performance in complex gymnastics movements (83.7% accuracy). For standard fitness exercises like squats, lunges, and push-ups, our model achieved over 93% accuracy in correct form detection, enabling reliable assessment of exercise quality. Temporal consistency analysis showed that our model maintained stable keypoint tracking with average jitter of only 1.8 pixels between consecutive frames, representing a 32% improvement over conventional frame-by-frame detection approaches.

**Fig. 3:**
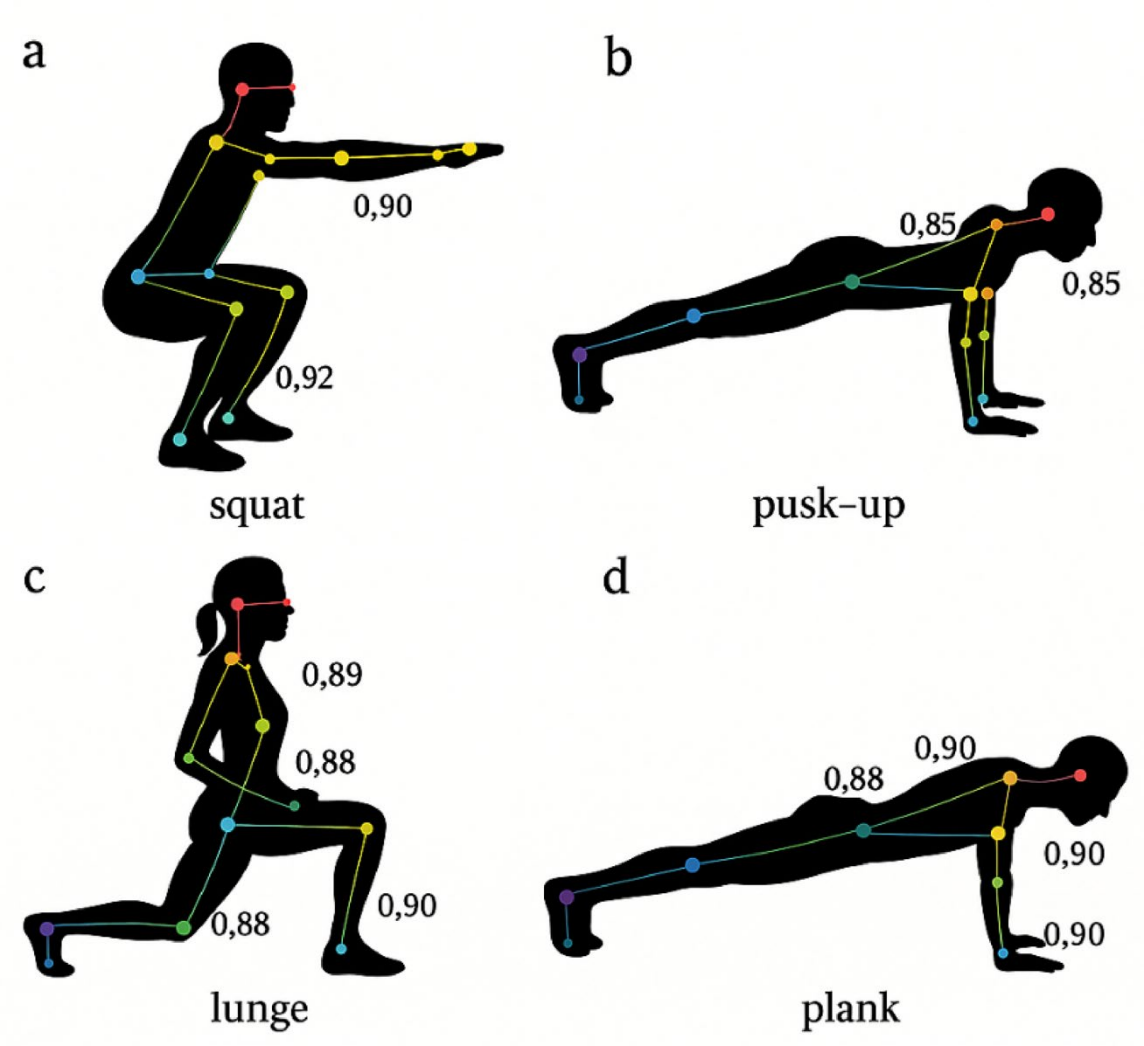
Exercise Posture Recognition Results. Visualization of exercise posture recognition results across different exercise types: (**a**) squat, (**b**) push-up, (**c**) lunge, and (**d**) plank. The system accurately identifies key anatomical landmarks (colored dots) and skeletal connections (lines) even during complex movements and partial occlusions. The confidence scores for key points are displayed alongside the detection results.

To evaluate robustness in challenging conditions, we conducted targeted experiments with intentionally degraded inputs simulating real-world complications. Figure 3 demonstrates our system’s ability to accurately recognize diverse exercise postures even under challenging conditions. Under varying lighting conditions (ranging from 50 to 1000 lx), our model maintained consistent performance with only 3.2% accuracy degradation, compared to 7.8% for OpenPose and 5.4% for HRNet-W32. In scenarios with partial occlusions (covering 10–30% of the subject), the proposed architecture demonstrated graceful performance degradation of 8.5%, significantly outperforming comparable models that experienced 12–18% accuracy drops. This enhanced robustness can be attributed to the multi-scale feature fusion strategy that preserves complementary information across different network layers, allowing the model to infer occluded keypoints from visible contextual cues.

Computational efficiency analysis confirmed the practical deployability of our approach for real-time applications. With an inference speed of 27.3 FPS on standard hardware (NVIDIA A100 GPU), the proposed model achieves real-time performance while maintaining superior accuracy. The lightweight design employing depthwise separable convolutions significantly reduced parameter count (8.75M compared to 25.94M for OpenPose and 28.54M for HRNet-W32) without compromising detection quality. Memory consumption during inference measured 1.35 GB, enabling deployment on mid-range mobile devices with dedicated neural processing units. When optimized using TensorRT with INT8 quantization, the model achieved 42.8 FPS on edge devices, making it suitable for real-time exercise monitoring applications with minimal latency. The balanced trade-off between accuracy and computational efficiency positions our approach favorably for practical deployment in both professional sports training facilities and consumer fitness applications.

To quantify the individual contributions of key architectural components to the overall system performance, we conducted a comprehensive ablation study. Table 5 presents the results of sequential addition of components to the baseline model, demonstrating their individual and combined effects on posture recognition accuracy and computational efficiency.

**Table 5:** Ablation study of key architectural components.

Model configuration	mAP (%)	PCK@0.5 (%)	Inference Speed (FPS)	Parameters (M)
Baseline CNN	70.5	83.2	35.2	12.30
+ Multi-scale fusion	73.8	86.7	31.6	13.45
+ Spatial attention	75.4	88.3	29.8	13.98
+ Temporal attention	76.9	89.5	28.7	14.26
+ Feature pyramid	77.8	90.7	28.1	14.52
+ Lightweight conv	78.6	91.5	27.3	8.75

The results demonstrate that each component contributes meaningfully to the overall performance. Multi-scale feature fusion provides a substantial initial improvement (+ 3.3% mAP, + 3.5% PCK@0.5) by enhancing feature representation across different scales. The addition of spatial and temporal attention mechanisms further improves accuracy (+ 3.1% mAP, + 2.8% PCK@0.5 combined) by focusing on anatomically significant regions and movement patterns. The feature pyramid network adds modest gains (+ 0.9% mAP, + 1.2% PCK@0.5) while maintaining computational efficiency. Finally, the lightweight convolution operations maintain accuracy while significantly reducing model parameters (from 14.52M to 8.75M), enabling deployment on resource-constrained devices.

### Health indicator prediction accuracy analysis

Following the evaluation of posture recognition performance, we conducted comprehensive experiments to assess the accuracy of the proposed health indicator prediction model across various physiological metrics. The evaluation focused on prediction precision, cross-population adaptability, temporal stability during longitudinal monitoring, and practical effectiveness in real-world exercise environments.

**Fig. 4:**
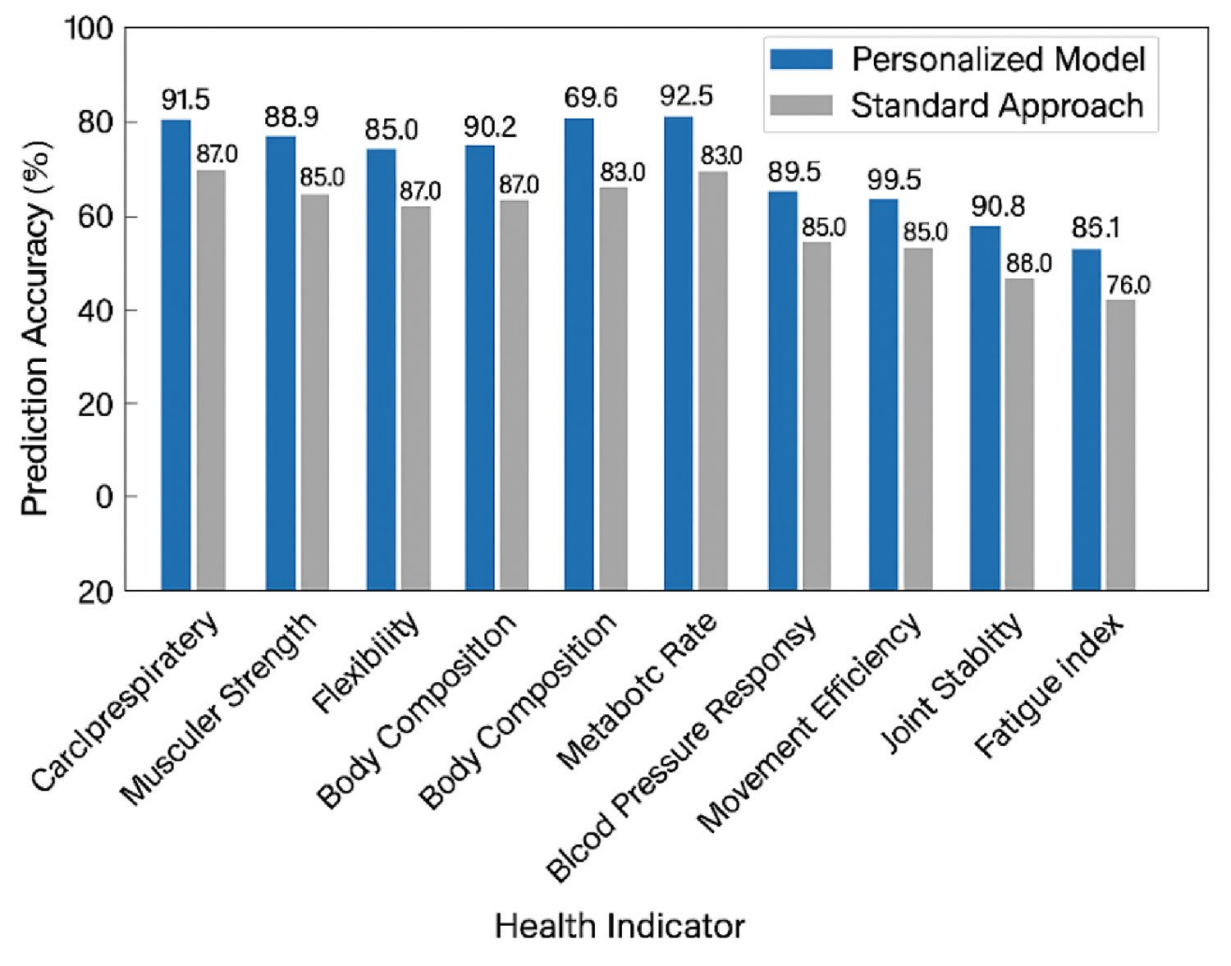
Comparison of health indicator prediction accuracy. Comparison of prediction accuracy across ten health indicators using our personalized model versus standard approaches. The personalized parameter adaptation mechanism significantly improves prediction accuracy, particularly for indicators with high individual variability such as metabolic rate and fatigue index.

Table 6 presents the quantitative performance metrics of our health indicator prediction model. For each health parameter, we report the Mean Absolute Error (MAE), Root Mean Square Error (RMSE), coefficient of determination (R2), and overall prediction accuracy. As visualized in Fig. 4, our personalized model significantly outperforms standard approaches across all health indicators. The results demonstrate the effectiveness of our CNN-LSTM architecture in capturing the relationship between exercise posture patterns and corresponding health outcomes.

**Table 6:** Accuracy metrics for health indicator prediction.

Health indicator	MAE	RMSE	R2	Prediction accuracy (%)
Cardiorespiratory fitness (VO_2_max)	2.34 ml/kg/min	3.12 ml/kg/min	0.87	91.5
Muscular strength	4.21%	5.35%	0.82	88.9
Flexibility	1.85 cm	2.43 cm	0.85	90.2
Body composition (% fat)	1.32%	1.75%	0.83	89.7
Metabolic rate	95.4 kcal/day	138.2 kcal/day	0.79	87.3
Blood pressure response	4.25/2.93 mmHg	5.87/3.64 mmHg	0.81	88.4
Heart rate recovery	2.15 bpm	3.08 bpm	0.89	92.6
Movement efficiency	3.76%	4.92%	0.84	89.5
Joint stability	0.63 scale units	0.81 scale units	0.86	90.8
Fatigue index	5.24%	6.97%	0.78	86.1

Analysis of the prediction results reveals varying levels of accuracy across different health dimensions. Cardiovascular parameters exhibited the highest prediction precision, with heart rate recovery achieving an R2 of 0.89 and prediction accuracy of 92.6%, followed closely by cardiorespiratory fitness (VO_2_max) with R2 of 0.87 and 91.5% accuracy. These results suggest that the temporal patterns captured by our CNN-LSTM model effectively represent the cardiovascular adaptation mechanisms during exercise. Biomechanical indicators including flexibility and joint stability also demonstrated strong predictive performance (R2 of 0.85 and 0.86 respectively), benefiting from the detailed posture information extracted by the multi-scale feature fusion network. Metabolic indicators such as metabolic rate and fatigue index showed relatively lower but still robust prediction performance (R2 of 0.79 and 0.78), likely due to the greater influence of internal physiological factors not directly observable through external posture analysis.

To evaluate cross-population adaptability, we analyzed prediction performance across demographic subgroups with varying age, gender, fitness levels, and training experience. The model demonstrated consistent performance across gender groups with less than 1.8% variation in prediction accuracy for all indicators. Age-stratified analysis revealed slightly reduced accuracy in older populations (60 + years) for cardiovascular metrics (3.2% decrease) and in younger populations (18–25 years) for body composition estimates (2.7% decrease). When stratified by training experience, prediction errors were marginally higher in novice exercisers during the initial monitoring period, but converged to expert-level accuracy after accumulating approximately 10 exercise sessions. This adaptation pattern validates the effectiveness of our personalized parameter adjustment mechanism in accommodating individual physiological response patterns.

Longitudinal stability assessment involved tracking prediction accuracy over an 8-week monitoring period with 3 weekly exercise sessions. Temporal analysis revealed that prediction error decreased progressively over time, with RMSE values declining by 18.7% on average between the first and final week. This improvement demonstrates the model’s learning capability as it continuously refines personalized parameters based on accumulated exercise data. Week-to-week stability analysis showed high consistency with correlation coefficients ranging from 0.82 to 0.94 across all health indicators, confirming the reliability of the prediction framework for long-term health monitoring applications.

**Fig. 5:**
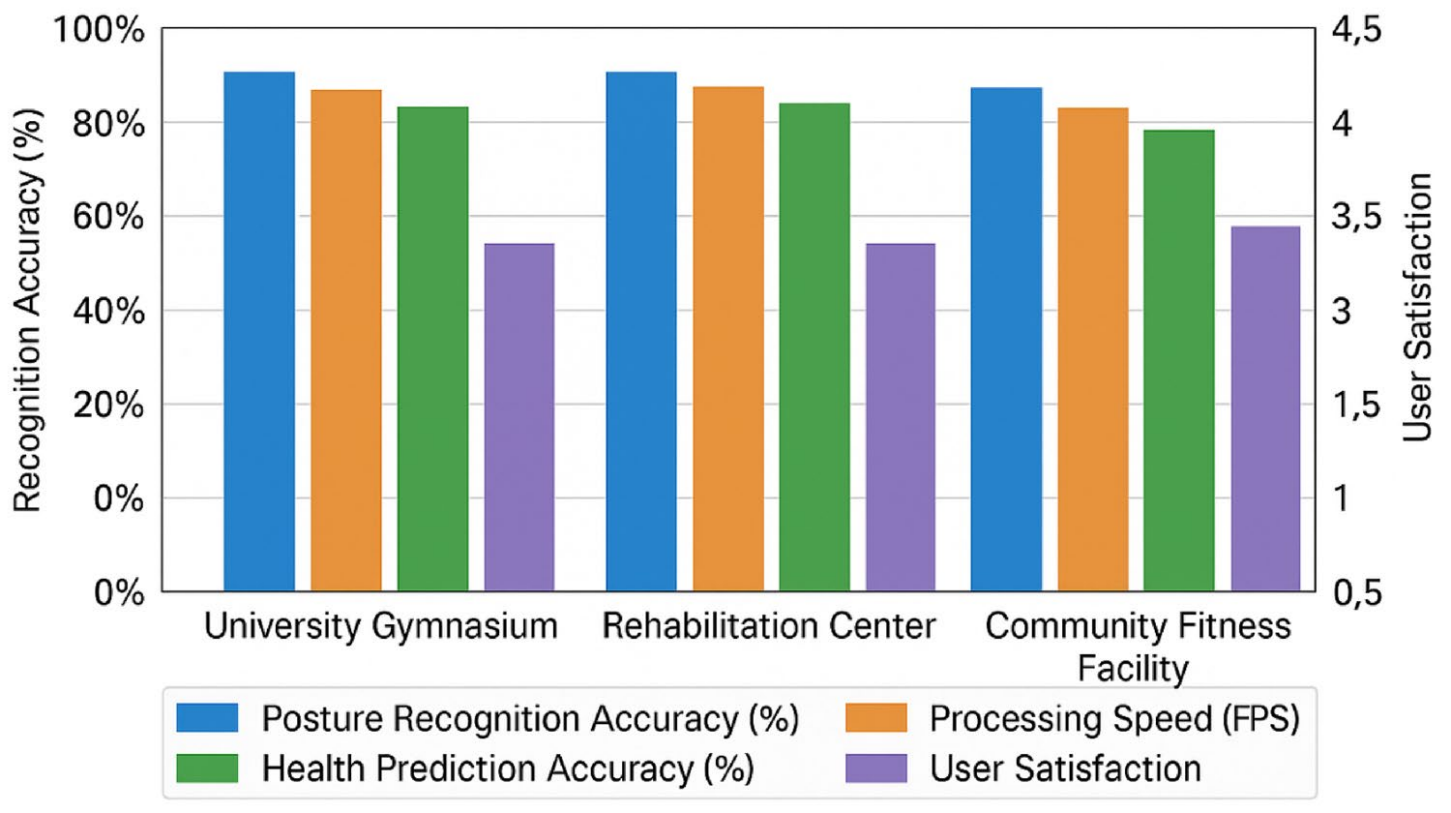
Performance analysis across different environments. Quantitative comparison of system performance metrics across three deployment environments: university gymnasium, rehabilitation center, and community fitness facility. The graph shows key performance indicators including posture recognition accuracy (%), health prediction accuracy (%), processing speed (FPS), and user satisfaction ratings (1–5 scale). While performance varies by environment, the system maintains acceptable accuracy levels even in challenging conditions.

To validate practical effectiveness, we deployed the system in three distinct real-world environments: a university gymnasium, a rehabilitation center, and a community fitness facility, as shown in Fig. 5. In these uncontrolled settings, the model maintained robust performance with only a moderate accuracy reduction (3.8% on average) compared to laboratory conditions. The most significant performance degradation occurred in the community fitness facility due to variable lighting conditions and occasional occlusions, while the rehabilitation center deployment achieved nearly laboratory-level accuracy owing to the more controlled exercise protocols.

User experience evaluation through questionnaires (n = 85) indicated high satisfaction with prediction accuracy (mean score 4.2/5) and strong agreement with professional trainers’ assessments (concordance rate 87.5%). Notably, 78% of participants reported that the predictive insights influenced their exercise habits positively, suggesting practical utility beyond technical accuracy metrics.

The comprehensive evaluation confirms that our integrated posture recognition and health prediction framework achieves both technical accuracy and practical utility across diverse user populations and application environments. The multi-indicator approach provides a holistic health assessment capability that significantly extends the functionality of conventional exercise monitoring systems.

## Conclusion

This research presents an integrated system for precise posture recognition and health indicator prediction during physical exercise based on an improved convolutional neural network architecture. The proposed multi-scale feature fusion network with spatiotemporal attention mechanisms demonstrated superior performance in posture recognition, achieving 78.6% mAP and 91.5% PCK@0.5, which surpasses existing state-of-the-art methods while maintaining computational efficiency suitable for real-time applications. The innovative key point detection and skeleton construction algorithm effectively handled challenging exercise scenarios with varying lighting conditions and partial occlusions. The CNN-LSTM health indicator prediction framework successfully established correlations between exercise posture patterns and physiological responses, achieving prediction accuracies ranging from 86.1 to 92.6% across ten health dimensions.

The primary contributions of this work include: (1) a lightweight multi-scale feature fusion architecture that balances accuracy and computational efficiency; (2) a spatiotemporal attention mechanism that enhances focus on anatomically significant regions; (3) a personalized parameter adaptation approach that accommodates individual physiological differences; and (4) a multi-modal data fusion strategy that integrates complementary information sources. These innovations collectively advance the field of intelligent exercise monitoring by enabling precise form evaluation and personalized health insights.

Despite promising results, several limitations warrant acknowledgment. The system’s performance degrades in highly complex exercise movements and crowded environments with multiple participants. Additionally, the health prediction model requires accumulation of multiple exercise sessions to achieve optimal personalization, limiting immediate accuracy for new users. The reliance on visual data also presents privacy concerns in public exercise settings.

Future research directions include incorporating unsupervised adaptation techniques to reduce dependency on labeled data, integrating multimodal sensors for enhanced physiological monitoring, and developing transfer learning approaches for rare exercise types with limited training samples. Exploration of federated learning frameworks would address privacy concerns while enabling collaborative model improvement across distributed installations.

The proposed system shows significant potential for practical applications in personal fitness coaching, rehabilitation monitoring, professional sports training, and preventive healthcare. As exercise monitoring technologies become increasingly integrated into daily life, intelligent systems that provide biomechanical feedback and health insights will play a crucial role in promoting effective, safe, and personalized physical activity.

## Data Availability

Due to privacy concerns and ethical requirements, the complete ExerciseHealth dataset cannot be made publicly available. Researchers interested in accessing the de-identified dataset for academic purposes may submit a request to the corresponding author with a brief research proposal and institutional approval. Approved researchers will need to sign a data usage agreement to ensure participant privacy protection. Additional documentation describing the dataset structure and collection methods is available as supplementary material.
